# Manual of General Medical Technology, Including Prescription Writing

**Published:** 1895-12

**Authors:** 


Manual of General Medical Technology, Including Prescription Writing. By Edward Curtis, A. M., M. D., Emeritus Professor of Materia Medica and Therapeutics, College of Physicians and Surgeons, Medical Department of Columbia College, in the City of New York. Third Edition, conforming to the U. S. Pharmacopeia of 1890. Pocket size (Wood's Pocket Manual Series), 245 pages. Price, $1.00. William Wood & Company, Publishers, 43, 45, 47 East 10th Street, New York City.

This little manual has become very popular, having reached a third edition within six years. It contains a vast amount of useful information, and in this edition it has been made to conform to the latest revision of the United States Pharmacopeia. It will be particularly serviceable to the beginner in medical practice.

